# Association between dietary intake of n‐3 polyunsaturated fatty acids and risk of colorectal cancer in the Japanese population: The Japan Collaborative Cohort Study

**DOI:** 10.1002/cam4.5098

**Published:** 2022-08-10

**Authors:** Ayako Kato, Chika Okada, Ehab S. Eshak, Hiroyasu Iso, Akiko Tamakoshi, the JACC Study Group

**Affiliations:** ^1^ Public Health, Department of Social Medicine Osaka University Graduate School of Medicine Osaka Japan; ^2^ Department of Public Health, Faculty of Medicine Minia University Minia Egypt; ^3^ Department of Public Health Hokkaido University Faculty of Medicine Sapporo Japan

**Keywords:** alpha‐linolenic acid, colorectal cancer, Japan, n‐3 polyunsaturated fatty acid, prospective study

## Abstract

**Background:**

Epidemiological studies of the dietary intake of specific n‐3 polyunsaturated fatty acids (PUFA) and anatomical subsite‐specific colorectal cancer (CRC) are limited. We examined the prospective associations of total n‐3 PUFA, marine‐derived n‐3 PUFA [combined eicosapentaenoic acid (EPA), docosapentaenoic acid (DPA), and docosahexaenoic acid (DHA)], and alpha‐linolenic acid (ALA) as plant‐derived n‐3 PUFA with the risk of CRC by subsite in the Japan Collaborative Cohort Study.

**Methods:**

The participants completed a self‐administered food frequency questionnaire and had no prior history of CRC. Cox proportional hazards model was used to determine the associations between n‐3 PUFAs intake and CRC risk overall and by anatomical subsite.

**Results:**

During the median 13.8‐year follow‐up period, 699 of the 42,536 participants aged 40–79 years developed incident CRC. An inverse association was found between dietary ALA intake and the risk of distal colon cancer; the multivariable hazard ratios and 95% confidence intervals for the highest quartiles (Q4) were 0.41 (0.21–0.81; *p* trend = 0.01) compared with the lowest quartiles (Q1). Marine n‐3 PUFA intake was not associated with CRC risk in the overall or anatomical subsite‐specific analyses.

**Conclusion:**

Our findings suggest that higher ALA intake may be beneficial for lowering the risk of distal colon cancer.

## 1 INTRODUCTION

Colorectal cancer (CRC) is the third most common cancer worldwide, followed by breast and lung cancer.^1^ The incidence of CRC has been decreasing in some countries,^2^, ^3^, ^4^ but not in Japan.^5^, ^6^ In 2017, 153,193 patients were diagnosed with CRC, which became the second most common cause of cancer‐related deaths in 2019 (13.7%) in Japan.^5^ Diet is among the established lifestyle factors affecting the risk of CRC, which also include smoking, alcohol consumption, obesity, and physical inactivity.^7^, ^8^

N‐3 polyunsaturated fatty acids (PUFAs) are fatty acids containing more than one double bond in their chemical structure, with the first double bond occurring on the third carbon from the methyl carbon end.^9^ According to the length of the carbon chain, n‐3 PUFAs with 18‐carbon atoms include alpha‐linolenic acid (ALA), which is derived mainly from plant oils, and n‐3 PUFAs with 20 or more carbon atoms include eicosapentaenoic acid (EPA), docosapentaenoic acid (DPA), and docosahexaenoic acid (DHA), which are derived from marine oils.^9^

The anti‐inflammatory and anticarcinogenic effects of n‐3 PUFAs support the hypothesis that n‐3 PUFAs could protect against cancer risk, including CRC.^10^ Recently, a large European cohort study on marine‐derived n‐3 PUFAs intake and CRC risk was conducted.^11^ Subsequently, a meta‐analysis reported that high blood n‐3 PUFA levels decreased CRC risk, and dietary n‐3 PUFA intake tended to be inversely associated with CRC risk.^12^ In that meta‐analysis, dietary ALA intake was not associated with CRC risk. The evidence on the effect of plant‐derived n‐3 PUFA (ALA) and anatomical subsite‐specific CRC is limited.^13^, ^14^

In this study, we aimed to determine the association between dietary n‐3 PUFAs intake and CRC risk in a large‐scale cohort study of cancer risk in Asia. We hypothesized different associations by anatomical subsite because the colon cancer risk varies by anatomical subsite due to different embryological and physiological features,^15^ and by specific n‐3 PUFAs due to different sources and chemical structures.^9^

## 2 MATERIALS AND METHODS

### 2.1 Study population

The Japan Collaborative Cohort Study for Evaluation of Cancer Risk (JACC) study was established in the late 1980s. Details of the JACC study are described in a previous publication.^16^ In summary, a sample of 110,585 residents from 45 administrative districts in Japan (46,395 men and 64,190 women) aged 40–79 years at baseline (between 1988 and 1990) participated in municipal health screening examinations. Participants completed self‐administered questionnaires containing questions related to their lifestyle and medical history. Individual informed consent was obtained in 36 areas, and group consent was obtained from the area leader (which was common in Japan before the Japanese government first established the ethical guidelines in 2002^17^) in the remaining nine areas prior to study participation. Depending on the guideline for Informed Consent in Epidemiologic Research,^18^ proposed by the research group funded by the Ministry of Health and Welfare, the appropriateness of the entire study was ethically reviewed and approved by the Ethical Board of the Nagoya University School of Medicine in 2000. Subsequently, the study was approved by the Ethical Board of Osaka University Graduate School of Medicine. In general, the JACC study was conducted in accordance with the Declaration of Helsinki.

Our analysis included only 65,042 participants because the data on cancer incidence were collected in 24 of the 45 areas from population‐based cancer registers or by reviewing the records of major local hospitals. Furthermore, 972 participants with a previous history of cancer at baseline and 21,534 participants without dietary data were excluded. The remaining 42,536 participants were included in the final analysis. The included participants were younger; had a lower prevalence of men, smoking, and a history of diabetes mellitus; and had a higher prevalence of drinking, walking, sedentary work, higher education, and family history of CRC than the participants excluded from the analysis (Supporting Information Table S1). Therefore, the CRC risk profile was unlikely to differ materially between the included and excluded participants.

### 2.2 Assessment of dietary intake

Food frequency questionnaire (FFQ) was used at baseline to obtain information on the intake of various food items. The FFQ included 33 food items with five multiple‐choice responses: almost never, 1–2 times/month, 1–2 times/week, 3–4 times/week, and almost daily. The responses were rated as follows: 0 (0/30 days), 0.05 (1.5/30 days), 0.214 (1.5/7 days), 0.5 (3.5/7 days), and 1.0 (30/30 days), respectively. The dietary intake of n‐3 PUFAs (total n‐3 PUFA, EPA, DPA, DHA, and ALA) was computed using the fifth edition of the Japan Food Tables to measure the amount of n‐3 PUFAs in each food. These contents were multiplied by the participants' scores for each food item, and then, the contents were summed. The portion size of the FFQ food items was estimated based on the intake reported by a subsample of the JACC study in a validation study.^19^ The n‐3 PUFA intakes from the FFQ [mean (SD) = 5.0 (0.9) mg/day] was validated against that estimated from 12‐day dietary records [mean (SD) = 10.0 (1.7) mg/day] in the validation study with a Spearman's rank correlation coefficient of 0.21 (*p* = 0.058).^19^ Data on supplementation (e.g., fish oil supplement) was not included in the survey; however, supplement use was uncommon among the Japanese population at baseline. The energy‐adjusted nutrient intake was computed using the residual method.^20^

### 2.3 Ascertainment of colorectal cancer

CRC cases were defined using the codes C18 to C20, as stated in the 10th revision of the International Statistical Classification of Diseases and Related Health Problems (ICD‐10). Colon cancer was defined using CRC code C18. Colon cancer was further classified by subsite: C18.0 to C18.5 for proximal colon cancer and C18.6 and 18.7 for distal colon cancer. The codes C18.8 and C18.9 were not used for further classification by subsite because they were overlapping sites and were unspecified by subsite. C19 and C20 codes were used to define rectal cancer.

### 2.4 Statistical analysis

The person‐years of follow‐up were computed from the baseline (1988–1990) to the first censoring, which included death, moving out of the study area, diagnosis of any cancer, or termination of the follow‐up. Participants who moved out of the study area were considered censored cases because the incidence rates after such moves could not be followed in our system. The follow‐up was terminated in 2009 in most areas. However, this treatment was discontinued in some areas before 2009. The lowest consumption category in quartiles (Q1) of each n‐3 PUFA energy‐adjusted intake for all participants was regarded as the reference. Multivariable‐adjusted hazard ratios (HRs) and 95% confidence intervals (CIs) for CRC, colon cancer, proximal colon cancer, distal colon cancer, and rectal cancer were calculated using Cox proportional hazards models. The confounding variables included age, sex, body mass index (BMI; <18.5 kg/m^2^, 18.5 to 24.9 kg/m^2^, or ≥25.0 kg/ m^2^), smoking status (never smoker, former smoker, current smoker of 1–19 cigarettes/day, or current smoker of ≥20 cigarettes/day), alcohol intake (never drinker, former drinker, current drinker of <2 Japanese drinks [46 g ethanol]/day, or of ≥2 Japanese drinks/day), walking duration (walking <30 min/day or ≥30 min/day), sports duration (sports <1 h/week or ≥1 h/week), education (attended school at ages ≤18 years or >18 years), sedentary work (yes or no), family CRC history in parents or siblings (yes or no), diabetes history (yes or no), and quartiles of energy, calcium, iron, saturated fatty acids, and fiber intake. Missing values for all covariates were grouped into an additional category and included in the model. In the sensitivity analyses, the HRs for any observed significant associations were calculated after excluding participants who had been diagnosed with CRC within the first 3, 5, or 10 years of follow‐up. SAS 9.4 (SAS Institute Inc., Cary, NC, USA) was used to perform all analyses. Statistical significance was set at *p*‐value <0.05.

## 3 RESULTS

Table 1 presents the baseline characteristics of the participants according to quartiles of total n‐3 PUFA, marine n‐3 PUFA, and ALA intake. The mean intake was 927 mg/day in the lowest quartile (Q1) and 2143 mg/day in the highest quartile (Q4) for total n‐3 PUFA; 280 mg/day and 1031 mg/day Q4 for marine n‐3 PUFA; and 480 mg/day and 1113 mg/day for ALA, respectively. Total n‐3 PUFA intake was positively associated with age, walking, and the selected nutrient intakes and inversely associated with being men, smoking, drinking, and sedentary work. Similar trends were observed for marine n‐3 PUFA and ALA intakes.

**TABLE 1:** Risk characteristics of participants at baseline according to quartiles of total n‐3 polyunsaturated fatty acid, marine n‐3 polyunsaturated fatty acid, and alpha‐linolenic acid intake^a^

		Quartile of total n‐3 PUFA intake^b^							Quartile of marine n‐3 PUFA intake^b^ ^,^ ^c^								Quartile of ALA intake^b^							
		Q1	Q2		Q3		Q4		Q1		Q2		Q3		Q4		Q1		Q2		Q3		Q4	
Dietary intake	(mg/day)	927±326	1202±312		1536±314		2143±439		280±119		428±124		638±153		1031±214		480±149		647±175		814±164		1113±243	
No. of participants		10,634	10,634		10,634		10,634		10,634		10,634		10,634		10,634		10,634		10,634		10,634		10,634	
Age	(year)	55.2±9.9	56.2±10.2		56.7±10.1		57.7±9.7		55.9±10.2		56.3±10.2		56.3±10.0		57.3±9.5		55.1±9.6		56.0±10.0		56.7±10.0		58.2±10.1	
Body mass index	(kg/m^2^)	22.8±3.1	22.8±2.9		22.7±3.0		22.9±3.6		22.8±3.1		22.8±3.0		22.8±3.3		22.9±3.2		22.9±3.1		22.7±3.2		22.7±3.0		22.9±3.4	
Men	(%)	54.8	36.9		33.6		30.2		51.7		37.3		34.2		32.3		53.2		38.1		33.6		30.1	
Current smoker	(%)	34.7	22.5		19.9		17.6		31.7		22.6		20.8		19.7		34.7		23.3		20.0		16.8	
Regular drinker (≥46 g ethanol/day)	(%)	55.3	40.2		37.1		33.0		50.6		39.7		37.7		37.8		56.5		42.4		36.8		30.0	
Daily walking time (≥30 min/day)	(%)	68.4	70.1		71.7		72.4		70.3		70.2		70.6		71.3		67.4		69.1		72.4		73.9	
Sports (≥1 h/week)	(%)	26.1	26.5		28.3		27.7		26.5		27.6		27.1		27.4		25.2		27.0		28.3		28.2	
Sedentary work	(%)	16.9	16.7		15.9		13.9		16.1		17.1		16.1		14.4		16.7		17.4		15.9		13.3	
Education (ages >18 years)	(%)	15.5	15.1		14.3		14.4		15.1		14.5		14.2		15.5		14.9		16.3		14.6		13.3	
History of diabetes mellitus	(%)	5.4	4.7		5.0		5.0		5.1		4.8		4.7		5.4		5.8		5.0		4.9		4.4	
Family history of colorectal cancer	(%)	1.5	1.6		1.4		1.3		1.4		1.5		1.3		1.5		1.5		1.6		1.4		1.3	
Energy	(kcal/day)	1588±472	1439±419		1482±410		1588±405		1598±452		1432±412		1487±439		1580±402		1580±474		1457±427		1476±395		1585±414	
Calcium intake	(mg/day)	381±141	431±138		480±137		552±145		421±151		435±146		466±147		521±152		367±136		432±133		481±131		564±143	
Iron intake	(mg/day)	576±194	655±190		744±191		875±212		649±217		662±209		723±216		815±224		554±183		647±182		746±177		901±201	
Saturated fatty acids intake	(mg/day)	870±349	937±342		1019±342		1168±375		915±367		946±352		1011±350		1121±375		864±342		951±336		1008±338		1170±390	
Total dietary fiber intake	(g/day)	10.6±3.3	11.3±3.3		12.4±3.3		14.1±3.5		11.8±3.6		11.4±3.5		12.1±3.5		13.2±3.6		10.0±3.1		11.1±3.0		12.5±2.9		14.9±3.3	

Abbreviations: ALA, alpha‐linolenic acid; PUFA, polyunsaturated fatty acid.

^a^ Mean ± standard deviation for continuous variables and percentages for categorical variables.

^b^ Energy‐adjusted quintiles using the residual nutrient model.

^c^ Marine n‐3 PUFA: EPA + DPA + DHA.

In the 585,644 person‐years of follow‐up (median years = 13.8) of the 42,536 participants, 699 patients developed CRC, including 462 with colon cancer (204 proximal colon, 187 distal colon cancer, and 71 overlapping or unspecified subsite cases) and 237 with rectal cancer.

Table 2 shows the HRs for the quartiles of the total n‐3 PUFA intake. No association was found between total n‐3 PUFA and CRC, colon cancer (both entire colon and colon by subsite), or rectal cancer.

**TABLE 2:** Hazard ratios and 95% confidence intervals of colorectal cancer incidence according to quartiles of total n‐3 polyunsaturated fatty acid intake

	Quartile of total n‐3 PUFA intake^a^							
	Q1	Q2		Q3		Q4		*p* for trend
No. of participants	10,634	10,634		10,634		10,634		
Person‐years	130,614	141,366		151,573		162,192		
Colorectal cancer								
No. of cases	157	174		184		184		
Age‐ and sex‐adjusted HR	1.00	1.08	(0.87–1.34)	1.07	(0.86–1.32)	0.99	(0.80–1.23)	0.85
Multivariable HR^b^	1.00	1.20	(0.95–1.52)	1.22	(0.95–1.57)	1.11	(0.84–1.46)	0.55
Colon cancer								
No. of cases	104	111		127		120		
Age‐ and sex‐adjusted HR	1.00	0.99	(0.76–1.30)	1.05	(0.80–1.36)	0.91	(0.69–1.19)	0.54
Multivariable HR^b^	1.00	1.16	(0.87–1.55)	1.29	(0.95–1.75)	1.11	(0.79–1.56)	0.51
Proximal colon cancer								
No. of cases	38	48		56		62		
Age‐ and sex‐adjusted HR	1.00	1.09	(0.71–1.67)	1.14	(0.75–1.74)	1.13	(0.75–1.71)	0.56
Multivariable HR^b^	1.00	1.25	(0.79–1.98)	1.42	(0.88–2.30)	1.40	(0.83–2.36)	0.22
Distal colon cancer								
No. of cases	56	42		52		37		
Age‐ and sex‐adjusted HR	1.00	0.77	(0.52–1.16)	0.90	(0.61–1.33)	0.61	(0.40–0.94)	0.06
Multivariable HR^b^	1.00	0.84	(0.55–1.30)	1.03	(0.66–1.60)	0.68	(0.40–1.16)	0.30
Rectal cancer								
No. of cases	53	63		57		64		
Age‐ and sex‐adjusted HR	1.00	1.27	(0.88–1.83)	1.10	(0.75–1.61)	1.18	(0.81–1.71)	0.59
Multivariable HR^b^	1.00	1.29	(0.87–1.92)	1.09	(0.71–1.69)	1.13	(0.70–1.80)	0.87

Abbreviations: HR, hazard ratio; PUFA, polyunsaturated fatty acid.

^a^ Energy‐adjusted quintiles using the residual nutrient model.

^b^ Multivariate HR adjusted for age, sex, area, BMI, smoking status, alcohol consumption, walking, sports, education, sedentary work, history of diabetes mellitus, family history of colorectal cancer, and quartiles of total energy, calcium, iron, saturated fatty acids, and fiber intake.

Marine n‐3 PUFA intake (combined with EPA, DPA, and DHA) was not associated with CRC at any anatomical subsite (Table 3).

**TABLE 3:** Hazard ratios and 95% confidence intervals of colorectal cancer incidence according to quartiles of marine n‐3 polyunsaturated fatty acid intake

	Quartile of marine n‐3 PUFA intake^a^							
	Q1	Q2		Q3		Q4		*p* for trend
No. of participants	10,634	10,634		10,634		10,634		
Person‐years	139,482	143,172		147,961		155,129		
Colorectal cancer								
No. of cases	171	173		162		193		
Age‐ and sex‐adjusted HR	1.00	1.05	(0.85–1.30)	0.97	(0.78–1.20)	1.09	(0.88–1.34)	0.62
Multivariable HR^b^	1.00	1.06	(0.86–1.32)	0.97	(0.77–1.21)	1.07	(0.86–1.34)	0.75
Colon cancer								
No. of cases	112	114		105		131		
Age‐ and sex‐adjusted HR	1.00	1.03	(0.79–1.33)	0.92	(0.70–1.20)	1.08	(0.83–1.39)	0.75
Multivariable HR^b^	1.00	1.03	(0.79–1.35)	0.94	(0.71–1.24)	1.09	(0.83–1.43)	0.69
Proximal colon cancer								
No. of cases	41	49		55		59		
Age‐ and sex‐adjusted HR	1.00	1.14	(0.75–1.73)	1.24	(0.82–1.86)	1.22	(0.82–1.83)	0.31
Multivariable HR^b^	1.00	1.17	(0.77–1.78)	1.28	(0.84–1.96)	1.25	(0.81–1.92)	0.30
Distal colon cancer								
No. of cases	54	50		33		50		
Age‐ and sex‐adjusted HR	1.00	1.01	(0.68–1.48)	0.65	(0.42–1.00)	0.95	(0.65–1.41)	0.41
Multivariable HR^b^	1.00	0.98	(0.66–1.46)	0.64	(0.41–1.00)	0.94	(0.62–1.43)	0.41
Rectal cancer								
No. of cases	59	59		57		62		
Age‐ and sex‐adjusted HR	1.00	1.11	(0.77–1.59)	1.06	(0.74–1.53)	1.10	(0.77–1.58)	0.66
Multivariable HR^b^	1.00	1.13	(0.78–1.63)	1.02	(0.70–1.49)	1.04	(0.70–1.53)	0.99

Abbreviations: HR, hazard ratio, PUFA, polyunsaturated fatty acid.

^a^ Marine n‐3 PUFA: EPA + DPA + DHA. Energy‐adjusted quintiles using the residual nutrient model.

^b^ Multivariate HR adjusted for age, sex, area, BMI, smoking status, alcohol consumption, walking, sports, education, sedentary work, history of diabetes mellitus, family history of colorectal cancer, and quartiles of total energy, calcium, iron, saturated fatty acids, and fiber intake.

Table 4 shows the HRs for the ALA intake quartiles. No associations were observed between ALA and overall CRC risk; however, ALA intake was inversely associated with distal colon cancer risk in a dose‐dependent manner [HR (95%CI) for Q2:0.93 (0.60–1.43), Q3:0.76 (0.44–1.29), and Q4:0.41 (0.21–0.81), *p* for trend = 0.01].

**TABLE 4:** Hazard ratios and 95% confidence intervals of colorectal cancer incidence according to quartiles of alpha‐linolenic acid intake

	Quartile of ALA intake^a^							
	Q1	Q2		Q3		Q4		*p* for trend
No. of participants	10,634	10,634		10,634		10,634		
Person‐years	127,096	139,111		154,211		165,326		
Colorectal cancer								
No. of cases	167	174		172		186		
Age‐ and sex‐adjusted HR	1.00	0.98	(0.80–1.22)	0.87	(0.70–1.08)	0.85	(0.69–1.06)	0.09
Multivariable HR^b^	1.00	1.11	(0.87–1.42)	1.06	(0.79–1.42)	0.97	(0.69–1.36)	0.75
Colon cancer								
No. of cases	113	116		117		116		
Age‐ and sex‐adjusted HR	1.00	0.93	(0.72–1.21)	0.82	(0.63–1.07)	0.72	(0.55–0.94)	0.01
Multivariable HR^b^	1.00	1.10	(0.81–1.48)	1.07	(0.75–1.52)	0.85	(0.56–1.29)	0.43
Proximal colon cancer								
No. of cases	39	49		52		64		
Age‐ and sex‐adjusted HR	1.00	1.07	(0.70–1.63)	0.96	(0.63–1.47)	1.02	(0.68–1.54)	0.94
Multivariable HR^b^	1.00	1.25	(0.77–2.01)	1.32	(0.75–2.31)	1.43	(0.76–2.69)	0.31
Distal colon cancer								
No. of cases	58	52		45		32		
Age‐ and sex‐adjusted HR	1.00	0.88	(0.60–1.28)	0.70	(0.47–1.04)	0.46	(0.30–0.72)	<0.001
Multivariable HR^b^	1.00	0.93	(0.60–1.43)	0.76	(0.44–1.29)	0.41	(0.21–0.81)	0.01
Rectal cancer								
No. of cases	54	58		55		70		
Age‐ and sex‐adjusted HR	1.00	1.10	(0.76–1.60)	0.98	(0.67–1.43)	1.17	(0.81–1.69)	0.52
Multivariable HR^b^	1.00	1.13	(0.74–1.74)	1.05	(0.63–1.73)	1.24	(0.70–2.19)	0.55

Abbreviations: HR, hazard ratio; ALA, alpha‐linolenic acid.

^a^ Energy‐adjusted quintiles using the residual nutrient model.

^b^ Multivariate HR adjusted for age, sex, area, BMI, smoking status, alcohol consumption, walking, sports, education, sedentary work, history of diabetes mellitus, family history of colorectal cancer, and quartiles of total energy, calcium, iron, saturated fatty acids, and fiber intake.

The results remained unchanged after excluding distal colon cancer cases diagnosed within 3, 5, or 10 years from the baseline; the HRs (95% CIs) after excluding cases that developed during the first 5 years from the baseline were as follows: Q2, 0.86 (0.48–1.53); Q3, 0.54 (0.26–1.10); and Q4, 0.23 (0.09–0.56) (*p* for trend = 0.001). The respective HRs (95% CIs) after excluding incident cases within the first 10 years were as follows: Q2, 1.02 (0.46–2.27); Q3, 0.67 (0.24–1.86); and Q4, 0.44 (0.12–1.54) (*p* for trend = 0.18) (Table 5).

**TABLE 5:** Hazard ratios and 95% confidence intervals of distal colon cancer incidence according to quartiles of alpha‐linolenic acid intake after exclusion of the incidence within 3, 5, and 10 years from the baseline

	Quartile of ALA intake^a^							
	Q1	Q2		Q3		Q4		*p* for trend
**Exclude 3 years**								
Dietary intake (mg/day)^b^	481 ± 180	648 ± 175		815 ± 164		1115 ± 244		
No. of participants	10,329	10,329		10,330		10,329		
Person‐years	126,787	139,022		153,807		164,258		
Distal colon cancer								
No. of cases	47	42		35		27		
Age‐ and sex‐adjusted HR	1.00	0.87	(0.57–1.32)	0.66	(0.42–1.03)	0.48	(0.29–0.77)	0.001
Multivariable HR^c^	1.00	0.87	(0.54–1.41)	0.65	(0.36–1.19)	0.38	(0.18–0.79)	0.01
**Exclude 5 years**								
Dietary intake (mg/day)^b^	497 ± 183	670 ± 176		832 ± 165		1129 ± 243		
No. of participants	9290	9290		9291		9290		
Person‐years	126,871	137,730		147,115		152,873		
Distal colon cancer								
No. of cases	31	33		27		17		
Age‐ and sex‐adjusted HR	1.00	1.02	(0.62–1.68)	0.79	(0.46–1.34)	0.48	(0.26–0.89)	0.01
Multivariable HR^c^	1.00	0.86	(0.48–1.53)	0.54	(0.26–1.10)	0.23	(0.09–0.56)	0.001
**Exclude 10 years**								
Dietary intake (mg/day)^b^	512 ± 185	698 ± 175		853 ± 165		1148 ± 243		
No. of participants	7823	7824		7824		7823		
Person‐years	126,286	130,669		133,727		135,411		
Distal colon cancer								
No. of cases	15	17		12		9		
Age‐ and sex‐adjusted HR	1.00	1.12	(0.56–2.27)	0.77	(0.35–1.69)	0.60	(0.26–1.40)	0.16
Multivariable HR^c^	1.00	1.02	(0.46–2.27)	0.67	(0.24–1.86)	0.44	(0.12–1.54)	0.18

Abbreviations: ALA, alpha‐linolenic acid; HR, hazard ratio.

^a^ Energy‐adjusted quintiles using the residual nutrient model.

^b^ Mean ± standard deviation of dietary intake.

^c^ Multivariate HR adjusted for age, sex, area, BMI, smoking status, alcohol consumption, walking, sports, education, sedentary work, history of diabetes mellitus, family history of colorectal cancer, and quartiles of total energy, calcium, iron, saturated fatty acids, and fiber intake.

No significant sex interaction was observed in analyses for Tables 2, 3, 4, 5.

## 4 DISCUSSION

In this large prospective study of a Japanese population, dietary intake of ALA was inversely associated with distal colon cancer risk in a dose‐dependent manner. After excluding the diagnosed distal colon cancer cases that occurred within 3, 5, and 10 years of baseline, the observed association remained unchanged. Dietary intakes of marine n‐3 PUFA and total n‐3 PUFA were, however, not associated with the risk of CRC at any subsites.

As only a small proportion of ALA can be transformed into EPA and DHA in the human body,^21^, ^22^ ALA per se may reduce the risk of distal colon cancer. The beneficial effects of ALA include reduction of aberrant crypt foci in the colon,^23^, ^24^, ^25^ induction of apoptosis,^26^, ^27^ and inhibition of proliferation, adhesion, and invasion of cancer cells.^28^ In rats, flaxseed oil, rich in ALA, inhibited inflammation and led to a reduction in tumorous lesions in the intestine and pancreas.^29^

Previous studies evaluating the association between ALA intake and CRC risk have yielded inconsistent findings. A previous Japanese prospective study showed no associations between dietary ALA intake and colon cancer risk; the multivariable relative risks (95% CIs) for the highest (median: 2760 mg/day in men and 2640 mg/day in women) quintiles of intake were 0.84 (0.56–1.28) (*p* for trend = 0.31) in men and 1.01 (0.65–1.57) (*p* for trend = 0.69) in women compared with that for the lowest (median: 1210 mg/day in men and 1350 mg/day in women) quintiles of intake.^13^ In that study, the association between ALA intake and the risk of distal colon cancer was analyzed only for invasive cancers, defined as tumors over the mucosal layer. A previous prospective study conducted in the United States (US) in 123,529 health professionals aged 40–75 years and nurses aged 30–55 years with 24 to 26 years of follow‐up also showed no association between dietary ALA intake and the risk of CRC, proximal colon, and distal colon cancer. The multivariable HRs (95% CIs) of CRC for the highest (≥1300 mg/day in men and ≥1200 mg/day in women) quartiles were 0.89 (0.70–1.13) (*p* for trend = 0.45) in men and 1.05 (0.86–1.29) (*p* for trend = 0.56) in women compared with those for the lowest (<900 mg/day both in men and in women) quartiles. The respective multivariable HRs (95% CIs) of proximal colon cancer were 0.81 (0.70–1.13) (*p* for trend = 0.45) in men and 1.04 (0.78–1.40) (*p* for trend = 0.89) in women. The respective multivariable HRs (95% CIs) of distal colon cancer were 1.15 (0.75–1.75) (*p* for trend = 0.39) in men and 1.18 (0.81–1.71) (*p* for trend = 0.25) in women.^14^

In contrast, some prospective studies investigating the association between dietary ALA intake and CRC risk reported a positive trend.^30^, ^31^, ^32^, ^33^ A 22‐year prospective study of 4967 Caucasians aged ≥55 years in Rotterdam, Netherlands, reported that dietary n‐3 PUFA from non‐fish sources was associated with an increased risk of CRC, with the HR (95% CI) for the highest (median:1400 mg/day) tertile of 1.76 (1.25–2.47) (*p* for trend = 0.001) compared with that for the lowest (median:700 mg/day) tertile.^30^ Another 6‐year follow‐up study on American individuals aged 50–74 years (97% of participants were white^34^) revealed a non‐significant positive association between dietary ALA intake and CRC risk among women. The multivariable HR (95% CI) for the highest (≥1190 mg/day) quartile of ALA was 1.38 (1.02–1.85) (*p* for trend = 0.13) compared with that for the lowest (<780 mg/day) quartile of ALA intake, and a non‐significant inverse association was observed between dietary ALA intake and the risk of CRC among men.^31^ The multivariable HR (95% CI) for the highest (≥1260 mg/day) quartile of ALA intake was 0.87 (0.66–1.14) (*p* for trend = 0.09) compared with that for the lowest (<820 mg/day) quartile of ALA intake among men.^31^ According to a 20‐year prospective study of 48,223 Swedish women aged 29–49 years, the multivariable HRs (95% CIs) of CRC and colon cancer for the highest (≥1160 mg/day) quartile of ALA intake were 1.17 (0.86–1.59) (*p* for trend = 0.112) and 0.95 (0.65–1.41) (*p* for trend = 0.833), respectively, compared with that for the lowest (<840 mg/day) quartile of ALA.^32^ An 11‐year prospective study of 59,986 Chinese men aged 40–74 years showed that the multivariable HRs (95% CIs) of CRC and colon cancer for the highest quartiles of ALA were 1.15 (0.92–1.43) (p for trend = 0.18) and 0.98 (0.74–1.31) (*p* for trend = 0.82), respectively, compared with that for the lowest quartile of ALA intake (amount of intake was not reported).^33^ In our study, the mean ALA intakes in the highest and lowest quartiles were 1175 and 525 mg/day among men and 1086 and 429 mg/day among women, respectively. The distribution of ALA intake in our study did not differ materially from those reported in previous studies.

The disparity between our study and previous studies that reported a positive trend may be due to the different dietary sources and different age distributions, shorter follow‐up period, and lack of information on the anatomical subsite of CRC in previous studies. For example, the primary sources of non‐marine n‐3 PUFAs associated with a higher risk of CRC^30^ in the Rotterdam study were butter and margarine.^35^ However, ALA is also present in flaxseed, canola, and soybean oils,^22^ among which soybeans are common in Japanese foods.^36^

Our results indicate an inverse association between dietary ALA intake and CRC risk only in patients with distal colon cancer. In rats, the incidence of aberrant crypt foci in the distal colon was higher than that in the proximal colon,^25^ and the number of aberrant crypt foci was reduced in the distal colon by flaxseed intake (rich in ALA) compared to that in the proximal colon.^23^, ^24^ In addition, the mucosa of the distal colon has a higher apoptotic index than that of the proximal colonic.^15^

In our study, no association was found between marine n‐3 PUFA intake and CRC risk, including in distal colon cancer. However, a US study conducted among health professionals and nurses suggested a positive association between marine n‐3 PUFA intake and the risk of distal colon cancer, with the multivariable HRs (95% CIs) in the highest quartiles of intake being 1.43 (0.97–2.11) among men and 1.36 (1.03–1.80) among women compared with those in the lowest quartiles of intake.^14^ The discrepancy between the findings from the US and our studies may be due to a large difference in the distribution of marine n‐3 PUFA intake; the highest quartiles among the US men (562 mg/day) and women (412 mg/day)^14^ corresponded to that between the second and third quartiles among Japanese men (473 and 710 mg/day) and that in the second quartile among Japanese women (402 mg/day) in our study. The high mean levels of marine n‐3 PUFA intake were observed across middle‐aged and older Japanese; 595 mg/day for the ages of 40–64 and 588 mg/day for the ages of 65–79 among men, and 603 mg/day and 568 mg/day, respectively, among women.

Moreover, no association was observed between marine n‐3 PUFA intake and risk of proximal colon cancer. Meanwhile, a previous Japanese study revealed a protective effect of marine n‐3 PUFA intake against invasive proximal colon cancer, with multivariable relative risks being 0.35 (0.14–0.88) among men and 0.59 (0.24–1.45) among women in the highest quintile compared with that in the lowest quintile.^13^ Furthermore, a recent report of the US health professionals and nurses' study revealed that a high marine n‐3 PUFA intake was associated with a lower risk of FOXP3 + T‐cell‐high CRC; the multivariable HRs (95% CIs) in the highest quartile were 0.57 (0.40–0.81, *p* for trend<0.001) for FOXP3 + T‐cell‐high CRC and 1.14 (0.81–1.60, *p* for trend = 0.77) for FOXP3 + T‐cell‐low CRC compared with those in the lowest quartile.^37^ The potency of marine n‐3 PUFA in proximal colon cancer may differ depending on the invasion levels and immune infiltrate of cancer; however, future research is needed to confirm this finding.

The strengths of our study were as follows: the prospective nature of the study, which avoided the occurrence of exposure recall bias; its large sample size with high follow‐up rates; and the informative analyses under sufficient quality of the cancer registry, which reduced the misclassification of outcomes. In addition, our study results were not affected by reverse causation because of the similar trend before and after excluding the early diagnosis of dietary ALA intake.

Our study had several limitations. First, although we adjusted for multiple potential confounders, some confounding and other unmeasured factors, such as the use of nonsteroidal anti‐inflammatory drugs, are suggested to reduce the CRC risk due to its anti‐inflammatory effect^38^; however, they remain unaccounted for. Second, dietary assessment using the FFQ is subject to recall bias, differences in the nutrient content of foods, and inability to capture the absorption and metabolism of PUFAs by the questionnaire. Furthermore, the data on n‐3 PUFAs intake obtained using the FFQ were available only at baseline; hence, it did not reflect the possible changes in the participants' dietary habits during the follow‐up period; however, such changes would have occurred randomly. Third, because we did not have information on the histological depth of CRC, the HRs depending on the invasion level could not be estimated.

In conclusion, our findings provide evidence that a high ALA intake is associated with a lower risk of distal colon cancer in a dose‐dependent manner. Further studies focusing on the health benefits of ALA intake on CRC risk are warranted.

## AUTHOR CONTRIBUTIONS

Ayako Kato, Chika Okada, and Hiroyasu Iso designed the study and methods of the analyses. Ayako Kato and Chika Okada analyzed the data. Ayako Kato drafted the manuscript. Ehab S Eshak, Hiroyasu Iso, and Akiko Tamakoshi provided a critical review of the content. Akiko Tamakoshi and Hiroyasu Iso coordinated the research. All authors approved the final manuscript.

## CONFLICT OF INTEREST

The authors declare that they have no competing interests.

## ETHICS STATEMENT

This study was approved by the Ethical Board of Nagoya University School of Medicine, Aichi, Japan, and Osaka University Graduate School of Medicine, Osaka, Japan.

## Supporting information


Table S1

## Data Availability

The raw data supporting the conclusions of this article will be made available by the authors, upon justified requests to the steering committee of the JACC study.
