# Supplementary material for: Association between dietary intake of n‐3 polyunsaturated fatty acids and risk of colorectal cancer in the Japanese population: The Japan Collaborative Cohort Study
Source: Cancer Med. 2022 Aug 10;12(4):4690–700. doi: 10.1002/cam4.5098 (PMC9972092; doi:10.1002/cam4.5098)
Supplement: Supplementary file 1 — Table S1 [file CAM4-12-4690-s001.docx]

Supplementary Table 1. Risk characteristics of the included and excluded participants due to lack of dietary data^1^.

|  |  | Included participants | | Excluded participants due to lack of dietary data | |
| --- | --- | --- | --- | --- | --- |
| No. of participants |  | 42536 | | 21534 | |
| Age | (year) | 56.5 | ±10 | 61.3 | ±9.9 |
| Body mass index | (kg/m^2^) | 22.8 | ±3.2 | 22.8 | ±3.5 |
| Men | (%) | 38.9 | | 44.7 | |
| Current smoker | (%) | 23.7 | | 27.6 | |
| Regular drinker (≥46g ethanol/day) | (%) | 41.5 | | 30.0 | |
| Daily Walking time (≥30min/day) | (%) | 70.6 | | 68.9 | |
| Sports (≥1hr/week) | (%) | 27.1 | | 27.9 | |
| Sedentary work | (%) | 15.9 | | 12.4 | |
| Education (ages >18 years) | (%) | 14.8 | | 12.5 | |
| History of diabetes mellitus | (%) | 5.0 | | 7.5 | |
| Family history of colorectal cancer | (%) | 1.5 | | 0.9 | |

^1^Mean ± standard deviation for continuous variables and percentages for categorical variables.
